# P-431. Mycobacterium abscessus Infection in Children - A 7 Year Experience in a Tertiary Paediatric Centre in Singapore

**DOI:** 10.1093/ofid/ofaf695.647

**Published:** 2026-01-11

**Authors:** Christopher Seow, Kai-Qian Kam, Valerie Xue Fen Seah, Karen Donceras Nadua, Chee Fu Yung, Jiahui Li, Koh Cheng Thoon, Natalie W Tan, Li Hwei Sng, Chia Yin Chong

**Affiliations:** KK Women's and Children's Hospital, Singapore, Singapore; KK Women's and Children's Hospital, Singapore, Singapore; KK Women's and Children's Hospital, Singapore, Singapore; KK Women's and Children's Hospital, Singapore, Singapore; KK Women's and Children's Hospital, Singapore, Singapore; KK Women's and Children's Hospital, Singapore, Singapore; KK Women's and Children's Hospital, Singapore, Singapore; KK Women's and Children's Hospital, Singapore, Singapore; Singapore General Hospital, Singapore, Not Applicable, Singapore; KK Women's and Children's Hospital, Singapore, Singapore

## Abstract

**Background:**

*Mycobacterium abscessus* is a fast-growing nontuberculous mycobacterium (NTM) and can lead to infections in both immunocompetent and immunocompromised children. Our study describes the clinical characteristics and treatment outcomes of *M. abscessus* infection in our population.

**Table 1:**
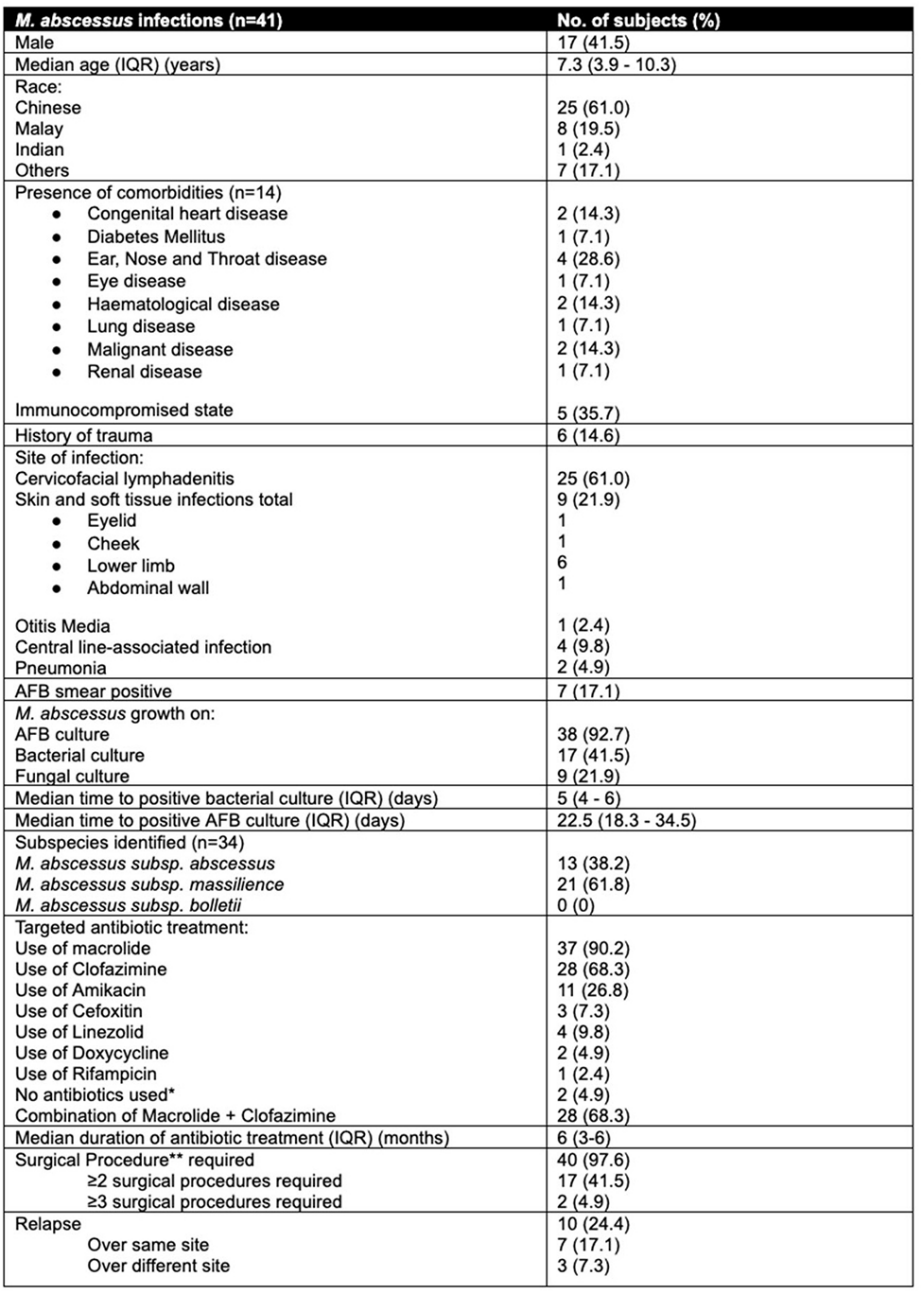
Summary of clinical characteristics, diagnosis, treatment and outcomes *antibiotics deemed not required after child underwent surgical procedure **includes removal of central line

**Table 2:**
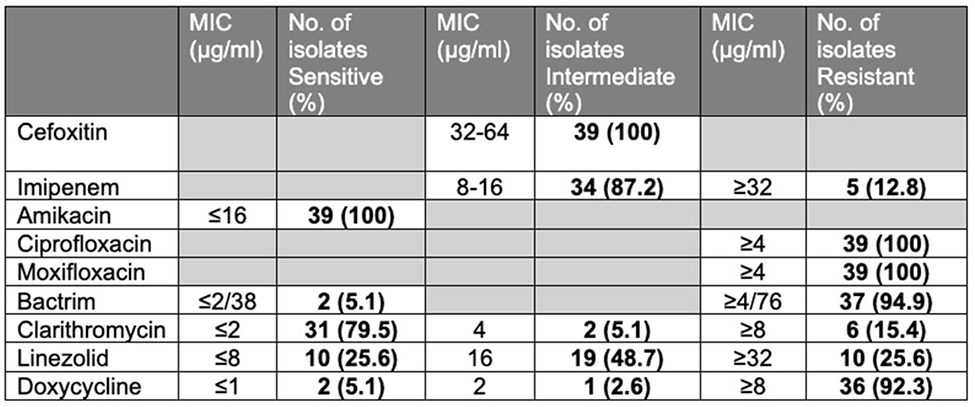
M. abscessus susceptibility* *Clofazimine susceptibility testing not performed locally

**Methods:**

From the laboratory database, we identified children aged 0-18 years with positive cultures for *M. abscessu*s over a 7-year-period (2018-2024) at KK Women’s and Children’s Hospital, a tertiary paediatric hospital in tropical Singapore. Clinical and microbiological data were collected.

**Table 3:**
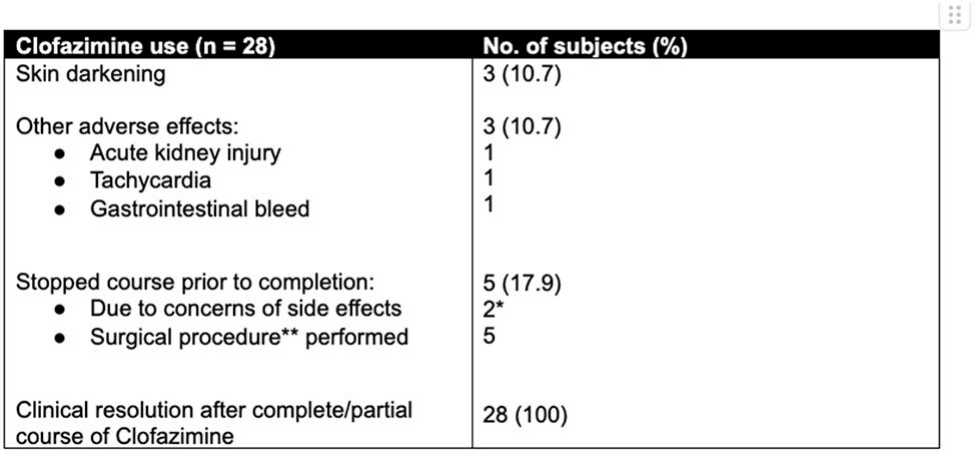
Adverse effects and treatment outcomes in children on clofazimine-based regimens *both had infected lines removed warranting no further treatment **includes removal of central line

**Results:**

There were 41 children with *M. abscessu*s infection. Median age was 7.3 years and 41.5% were males. Cervicofacial lymphadenitis was the most common clinical manifestation (n=25, 61.0%) followed by skin and soft tissue infections of the lower limbs (n=9, 21.9%) (Table 1). Median time to positive growth of *M. abscessus* on bacterial culture was significantly faster at 5 days compared to mycobacterial culture at 22.5 days (p< 0.001).

The majority of the isolates were sensitive to amikacin (100%) and clarithromycin (79.5%) (Table 2). All macrolide-resistant isolates were *subsp. abscessu*s. The most common treatment regime was the combination of a macrolide and clofazimine (68.3%). While clofazimine susceptibility was not performed locally, it was used based on 100% susceptibility from past publications. Side effects of Clofazamine were uncommon and not severe (Table 3). The median antimicrobial treatment duration was 6 months.

Relapse was seen in 10 cases (24.4%); 7 (17.1%) at the same site and 3 (7.3%) at different sites. Patients with relapse received 2.6 more months of antibiotics on average compared to those without relapse (p = 0.01).

Almost all (97.6%) patients required surgical procedures with 17 (41.5%) needing ≥2 surgeries. Complete resolution occurred in all patients and no mortality was observed.

**Conclusion:**

*Mycobacterium abscessus* infections can be challenging to treat in children requiring multiple surgical procedures and prolonged antimicrobial treatment. Source control with targeted antimicrobial therapy can lead to excellent outcomes.

**Disclosures:**

All Authors: No reported disclosures

